# The internal anatomy of titanosaur osteoderms from the Upper Cretaceous of Spain is compatible with a role in oogenesis

**DOI:** 10.1038/srep42035

**Published:** 2017-02-07

**Authors:** Daniel Vidal, Francisco Ortega, Francisco Gascó, Alejandro Serrano-Martínez, José Luis Sanz

**Affiliations:** 1 Grupo de Biología Evolutiva, UNED, Paseo Senda del Rey 9, 28040, Madrid, Spain; 2 Unidad de Paleontología, Universidad Autónoma de Madrid, C/Darwin 2 28049, Madrid, Spain

## Abstract

Dermal armor is one of the most intriguing features of some titanosaurs, the only sauropod dinosaurs that bore osteoderms. Some studies have revealed cavities of varying sizes inside some titanosaur osteoderms, interpreted as the result of bone remodeling for mineral mobilization. Several hypotheses have been proposed to explain the need for mineral mobilization in titanosaurs. However, rejecting those hypotheses was difficult with hitherto available evidence. The Upper Cretaceous site of Lo Hueco (Cuenca; Spain) has yielded one of the largest titanosaur osteoderm sets available. Observation of pre-existing breakages in the fossils and CT-scanning have revealed a predominant internal channel network for bulb and root osteoderms: most had a very compact spongy bone core, perfused by large longitudinal branching neurovascular canals. Only few osteoderms from the same bed, which was deposited in a single and short event, had areas with low-density spongy bone. This void-like low-density bone is always associated with internal channels. It is also present in osteoderms of different sizes. This scenario is best explained when considering that Lo Hueco titanosaurs might have used their osteoderms as a source of calcium that was mobilized during oogenesis, although other hypotheses cannot be completely ruled out.

The presence of dermal armor in some titanosaur sauropods was controversial until the publication of the osteoderms and other skeletal remains of *Saltasaurus loricatus* Bonaparte & Powell 1980 in the early 1980s^1^,^2^. At present titanosaur osteoderms are a well-established fact, although their internal structure, functions and amount of homoplasy on titanosaur evolution are still debated^3^,^4^,^5^. The number of osteoderms retrieved at each fossil-bearing locality is small. Fossil sites which have yielded several titanosaur individuals in various degrees of articulation have a low osteoderm/titanosaur individual ratio^4^,^6^,^7^,^8^ and some partially or mostly articulated titanosaur specimens have not been found in association with osteoderms. This has been interpreted as (i) a combination of taphonomic and collection bias against these dermal elements^3^, (ii) the hypothesis that most titanosaurs were lightly armored^3^,^4^,^7^ and (iii), in the case of no association with osteoderms^9^,^10^,^11^,^12^, that some species bore no armor at all.

Sauropod body plan is far from the paradigm of large armored tetrapods^13^. This, as well as the unique morphology of titanosaur osteoderms, have increased interest in understanding the function of titanosaur dermal armor^7^,^14^. Analyses of the internal microanatomy by means of histology and CT-scanning have revealed that osteoderms from different localities and taxa have very different internal compositions: a bulb and root osteoderm associated with an adult individual of *Rapetosaurus krausei* Curry Rogers & Forster 2001 has a large cavity inside^7^, whereas the scute morphotype osteoderms associated with *Saltasaurus loricatus* have no trace of such large voids^15^. The presence of large hollow spaces in titanosaur osteoderms has been interpreted to be the result of a physiological adaptation to different conditions (see Discussion) as mineral reservoirs^7^.

The Spanish sites of Armuña (Upper Campanian; Segovia, Spain^16^) and Lo Hueco (Campanian-Maastrichtian; Cuenca, Spain^8^) have yielded a relatively large sample of well-preserved titanosaur osteoderms. The sample from Lo Hueco was found within a large assemblage of at least two titanosaur morphotypes^17^ (the recently described *Lohuecotitan pandafilandi* Díez Díaz, Mocho, Páramo, Escaso, Marcos-Fernández, Sanz & Ortega^18^ as well as at least a second morphotype, still under description) and other fauna and flora^4^,^8^,^19^, with a large subsample coming from the same quarry bed. This set represents one of the best samples of osteoderms pertaining to several individuals from the same paleoenvironment in terms of preservation and number of specimens. Preliminary analyses studying the natural breakages of the osteoderms, CT-scan imaging as well as 3D reconstructions have shown areas of low density inside some of the osteoderms, whereas others are quite compact, with internal longitudinal and branching canals.

Here, the evidence from CT-scanning and osteoderms sections and fragments has revealed a prevailing pattern of neurovascular canals found in bulb and root titanosaur osteoderms, as well as the appearance of large areas of low-density bone in their cores associated with these canals. The presence of low-density bone among the sample from Lo Hueco, one of the largest available, allows testing the hypotheses proposed to explain mineral mobilization in titanosaur osteoderms.

## Results

**Figure 1:**
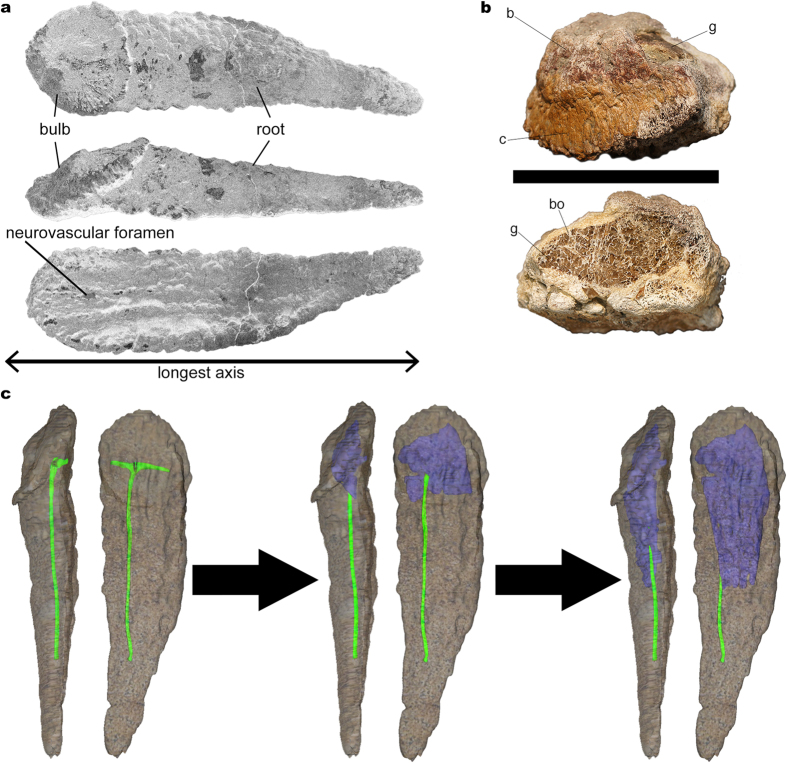
Titanosaur bulb and root osteoderms anatomy. (**a**) Basic bulb and root osteoderm anatomy shown in superficial (top), lateral (middle), and deep (bottom) views. (**b**) Fragmentary osteoderm HUE-08738 in lateral view (top), and its section (bottom), showing large trabeculae with chaotic pattern filled with gypsum within most of its volume, revealing the ultrastructure of titanosaur osteoderm internal large hollow spaces (reproduced from Vidal *et al*. 2014^3^). (**c**) 3D model of the internal canals based on the information revealed by fossils and a hypothetical transformation into large hollow spaces based on the evidence from HUE-00950 and HUE-02000. The low density bone appears in the largest volume, under the bulb region, and develops toward the root end. c = cingulum. b = bulb. bo = bone. g = gypsum.

**Figure 2:**
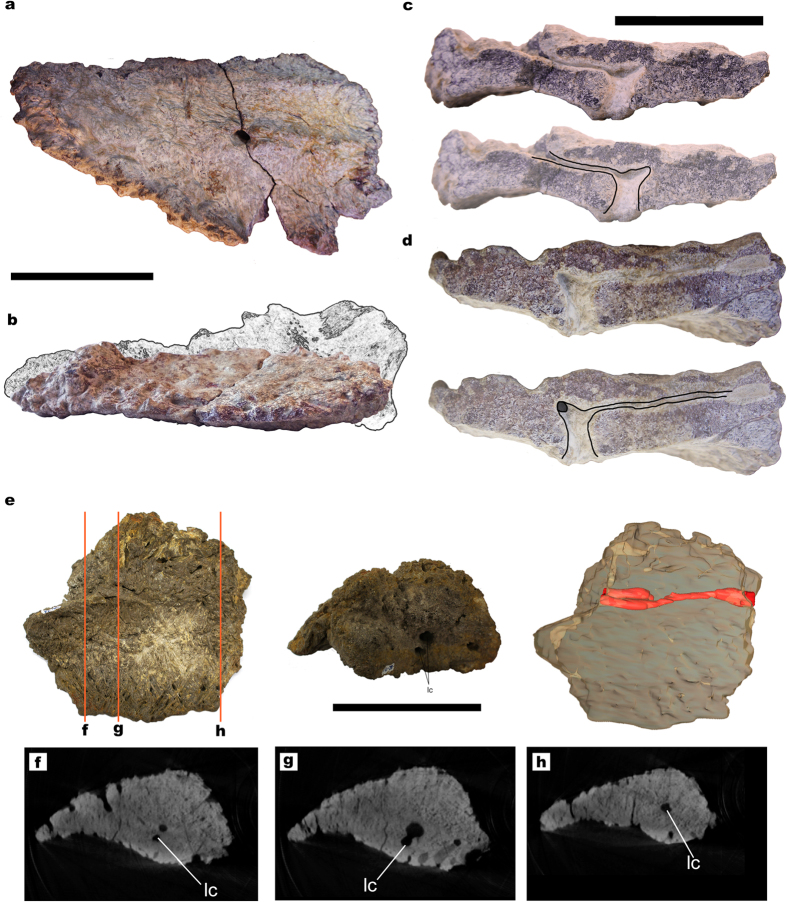
Titanosaur osteoderms with small canals and dense spongy bone. (**a**) HUE-02452 in deep view. Notice the fracture at the foramen level. (**b**) HUE-02452 in lateral view, with reconstructed missing portions (due to exposition to the elements, as it was collected *ex-situ* in surface) based on the morphological cline proposed by Vidal *et al*. 2014^3^. (**c**) Section of the smaller fragment of HUE-02452 (top) with interpretative drawing of the branching canal (below). (**d**) Section of the larger fragment of HUE-02452 (top) with interpretative drawing of the branching canal (below). Notice the branching toward the longitudinal canal, still filled with sediment. (**e**) UPUAM-13952 in deep (left), cross-section (middle) views and the 3D model (left) obtained from the CT-scan slices, with the longitudinal canal in red. (**f–h**) CT-scan slices at different positions of the osteoderm, in which the longitudinal canal can be seen. Scale for (**a**,**b**,**e**) = 100 mm. Scale for (**c**,**d**) = 50 mm. lc = longitudinal canal.

**Figure 3:**
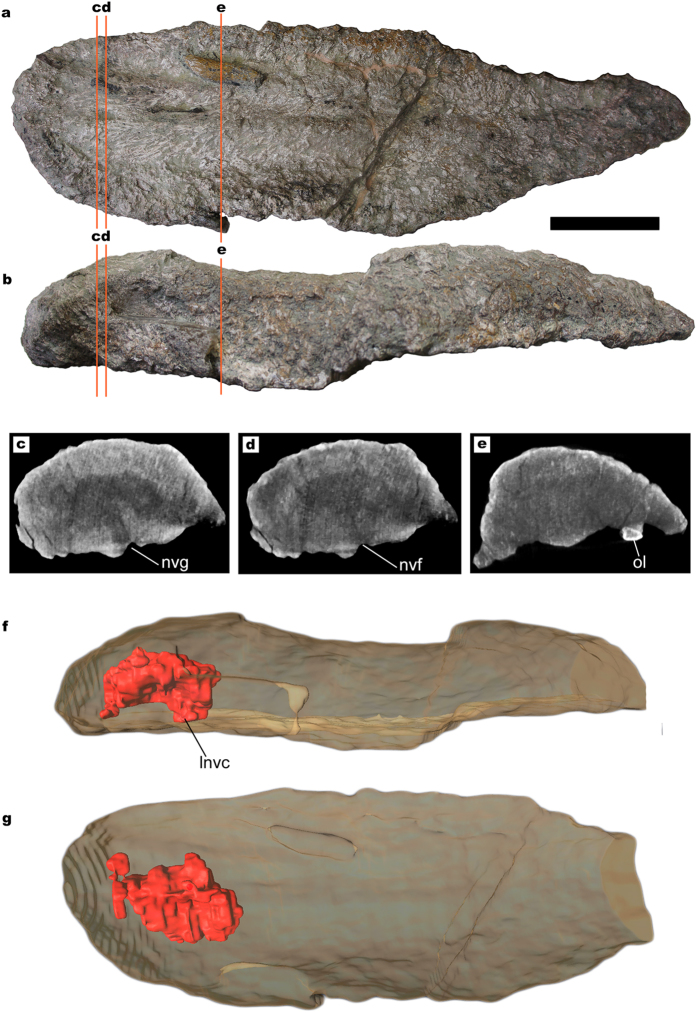
Titanosaur osteoderm with small internal cavities. (**a**) HUE-00950 in deep view. (**b**) HUE-00950 in lateral view. (**c–e**) CT-scan slices at different positions, where the lower density area and the neuro-vascular canal can be observed. (**f**) 3D model of HUE-00950 in lateral view, obtained from the CT-scan slices, with the void and canal in red. (**g**) 3D model of HUE-00950 in deep view. Scale = 100 mm. lnvc = longitudinal neurovascular canal. nvf = neurovascular foramen. nvg: neurovascular groove. ol = ossified ligament.

**Figure 4:**
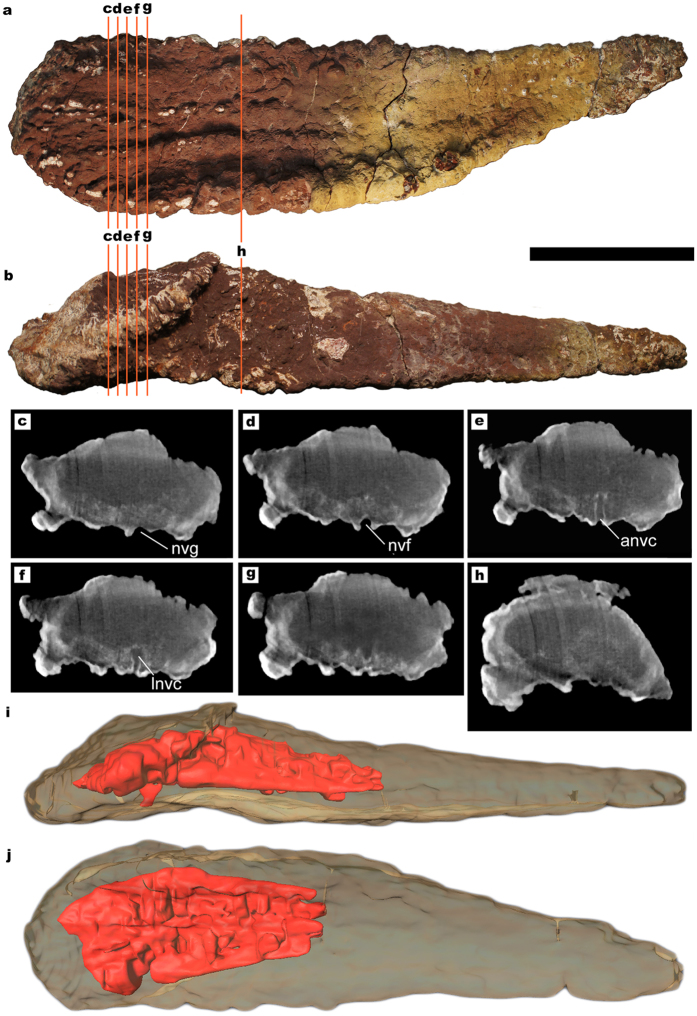
Titanosaur osteoderm with large internal cavities. (**a**) HUE-02000 in deep view. (**b**) HUE-02000 in lateral view. (**c–h**) CT-scan slices at different positions, where the lower density area and the neuro-vascular canal can be observed. Notice how the neurovascular canal walls are well differentiated by the iron mineral crust covering it, and that is denser than any material in the interior of the osteoderm. (**i**) 3D model of HUE-02000 in lateral view, obtained from the CT-scan slices, with the void and canal in red. (**j**) 3D model of HUE-02000 in deep view. Scale = 100 mm. anvc = ascending neurovascular canal. lnvc = longitudinal neurovascular canal. nvf = neurovascular foramen. nvg: neurovascular groove. ol = ossified ligament.

The anatomical terminology employed for titanosaur osteoderms is summarized in Fig. 1a. From 17 studied bulb and root specimens, eight had dense spongy bone (without large trabeculae or hollow spaces) with canals (Fig. 2), four had large hollow spaces in their cores (Figs 1b, 3 and 4), and five were too fragmentary to assess the presence or total absence of large hollow spaces. Eleven of these osteoderms come from the same bed in the quarry, G1 (Table 1). Among the specimens from the G1 bed sample, three osteoderms had hollow spaces, seven had dense spongy bone and one was too fragmented to assess its internal anatomy.

**Figure 5:**
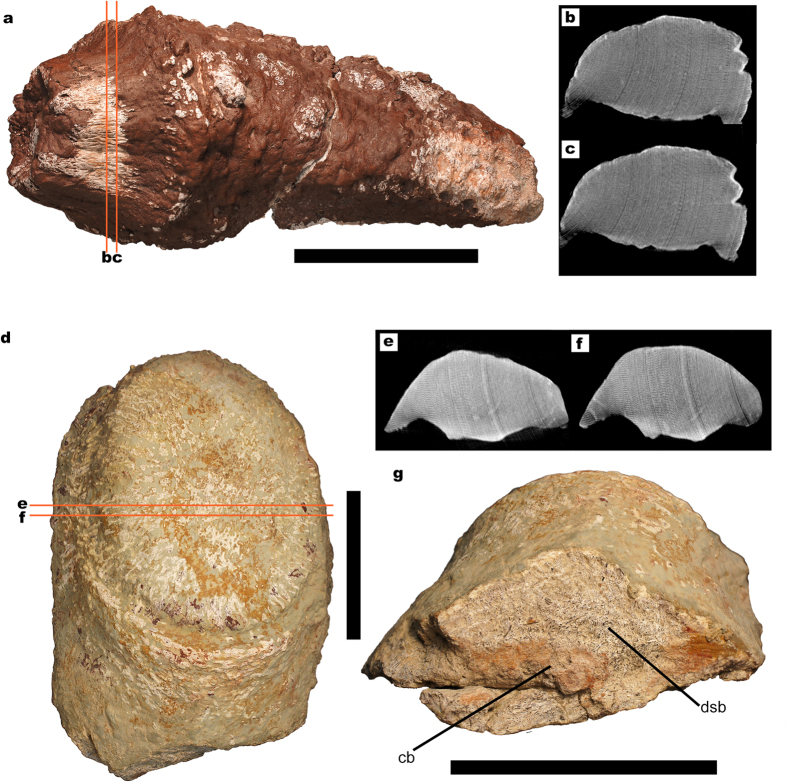
Titanosaur osteoderms without large internal cavities. (**a**) HUE-02326 in superficial view. (**b,c**) CT-scan slices at different positions, where no traces of the lower density bone can be observed. (**d**) HUE-02029 in superficial view. (**e–g**) CT-scan slices at different positions, where no traces of the lower density bone can be observed. (**f**) Root-end view of HUE-02029 showing a natural breakage, where most of the section is formed by very dense spongy bone (dsb). In some areas, the spongy bone has collapsed (cb) where longitudinal canals might have been present as in UPUAM-13952. All scales = 100 mm. cb = collapsed bone. dsp = dense spongy bone.

Most natural breakages revealed sections made of compact bone within the cortex and quite dense spongy bone in the core, with trabeculae filled by sediment without recognizable voids, reabsorption cavities or evidence of heavy and continuous remodeling, such as past cavities filled with newly deposited bone (Figs 2 and 5g). One breakage from a single specimen (HUE-08738, Fig. 1b) revealed a section with very large trabeculae and areas with very low-density spongy bone, with the voids filled with gypsum. This area of low-density bone is well delimited, cavity-like, with a very clear difference in bone density from the outer bone.

One osteoderm, whose superficial side is eroded (HUE-02452, Fig. 2a–d) shows a main canal through a breakage. It is connected with the large foramen located under the bulb in the deep side, and then it branches to much smaller canals. These branching canals appear to perfuse the osteoderm in deep-superficial direction from the neurovascular foramen, then branch laterally and in the antero-posterior axis (Fig. 2a–d, reconstruction in Fig. 1c). This osteoderm also has blind-ended canals of smaller diameter, which perfuse the osteoderm from the superficial or lateral sides and do not connect with the main canal. Another specimen from the site of Armuña (Segovia), broken at both ends of its longest axis (UPUAM-13952, Fig. 2e–h) has, in both sections, a canal parallel to the longest axis and blind ended canals identical to HUE-02452.

The CT-scans revealed some areas with a lower density inside the core in three of the scanned osteoderms, always in the bulb region (Figs 3 and 4). These low-density areas occupy mostly small volumes, well delimited, with no gradient of bone density toward the outer layers. However, one of the specimens shows a large volume of low-density bone (HUE-02000, Fig. 4). These lower density areas are connected with the large neurovascular foramen in the deep surface under the bulb in all cases by the same canal that connects with the neurovascular foramen in HUE-02452. They follow the internal network of neurovascular canals found in other osteoderms, but they are much larger than the internal canals seen in HUE-02452 and in UPUAM-13952. Given this, we interpret these lower density areas as low-density bone similar to the one in HUE-08738 (Fig. 1b). This low-density bone is associated with the neurovascular network of the osteoderm in all cases. Two of the scanned specimens, however, appeared to have a constant density in their ultrastructure, with no traces of canals or hollow spaces (Fig. 5).

Summing up, the analyzed bulb and root osteoderms had a large main canal, which perfused the osteoderm entering the neurovascular foramen in the deep side under the bulb. This canal then would branch in a cross pattern as seen in HUE-02452 and the CT-scans, reaching almost the end of the root as evidenced by UPUAM-13952. A hypothetical reconstruction of the complete channel based upon the existing evidence is presented in Fig. 1c.

## Discussion

As in extant crocodiles, titanosaur osteoderms could have had several functions: (i) the ornamentation in the bulb of these osteoderms suggests the presence of a keratin sheath, which might imply display or defensive function of some sort^20^,^21^; (ii) the well developed keels in the basal surface suggest the insertion of tendons and/or ligaments (there is an ossified tendon/ligament in the basal surface of HUE-00950; Fig. 3e), and some osteoderms being at least twice the length of a vertebra, imply a stiffening of the backbone. A similar function has been proposed for the armor of crocodiles^22^ and Thyreophora^23^; (iii) dermal ossicles, at present only found associated with *Saltasaurus loricatus*, might have given reinforcement to the integument^15^; (iv) a thermoregulatory function in titanosaur osteoderms has been considered, but ruled out due their low surface area/volume ratios^7^; and (v) the internal branching canals, the large trabeculae, the presence of Harvesian canals seen in histological sections and the hollow spaces associated with the branching canals (Figs 1c, 3 and 4) imply they could be a potential source of labile minerals^7^.

The first evidence of large hollow spaces in titanosaur osteoderms was a large osteoderm (FMNH PR 2342) associated with the ischia of a specimen of an adult-sized *Rapetosaurus*^7^, the largest cited specimen of this taxon. This osteoderm revealed a hollow space filled with sediment through breakage, and a CT-scan revealed a well defined cavity inside, occupying more than 50% of the volume of the osteoderm^7^. It is unclear from the description if there were small bony fragments or trabeculae inside the cavity or if it was empty except for the sediment that filled it, as in HUE-08738 (Fig. 1b). Other hollow osteoderms have been reported from the Maevarano Formation^7^, but it is unspecified whether they came from different sites or assemblages or not, if they are associated with skeletal material, what their sizes are or what the hollow/compact osteoderms ratio is among the sample. However, Curry-Rogers *et al*. determined this incidence of hollow osteoderms in the Maevarano titanosaurs precluded pathology as a cause for the cavities^7^. Histological studies of saltasaurine titanosaur osteoderms found evidence of vascularized endosteal bone, but could not determine whether it was pathologic or not^24^.

Carrano & D’Emic also cite the presence of a “crushed medullary cavity” in an osteoderm associated with *Alamosaurus sanjuanensis*, although it is impossible to tell whether or not it was hollow as in *Rapetosaurus*^25^. Other published titanosaur osteoderms sections reveal quite dense trabecular bone with canals and small reabsorption cavities (Fig. 2E in Dodson *et al*.^26^; Fig. 2a in Cerda & Powell^15^) and, sometimes, cancellous bone with large trabeculae (Fig. 8 in Cerda *et al*.^27^). Other titanosaur osteoderms, however, have not yielded evidence of very large hollow spaces inside them: (i) the scute morphotype osteoderms from *Saltasaurus loricatus* show only elongated canals connecting external, basal and lateral surfaces to the core as well as resorption cavities, but very much smaller than the ones in *Rapetosaurus*^15^; (ii) the scute morphotype osteoderms from Cinco Saltos (Argentina) show hollow spaces in their spongy bone cores (Fig. 8b–e in Cerda *et al*.^27^), but none as large as the ones in HUE-08738 (Fig. 1b); (iii) a bulb and root osteoderm from Salitral Moreno (Argentina) was found to be equally dense to a CT-scanning (Fig. 8d in Cerda *et al*.^27^); and (iv) a bulb fragment from a bulb and root osteoderm from Salitral Moreno (Argentina) showed evidence of internal canals through natural breakages but no evidence of low density bone (Fig. 8h–i in Cerda *et al*.^27^).

The sample from Lo Hueco introduces new evidence of large hollow spaces inside titanosaur osteoderms, comparable in volume to those found in *Rapetosaurus* osteoderm FMNH PR 2342 (Figs 1b and 4). The incidence of large hollow spaces among the sample from Lo Hueco, however, is low: only three osteoderms out of ten from the same bed have large hollow spaces. One of the osteoderms, HUE-00950, has a hollow cavity under the bulb (Fig. 3) and is associated with the partially articulated remains of the titanosaur individual HUE-EC-11 (Fig. 10 in Vidal *et al*.^4^ and Supplementary Fig. 1), which is not assignable to *Lohuecotitan* in a preliminary analysis. With the available evidence, the hitherto proposed hypotheses to explain the existence of hollow spaces inside titanosaur osteoderms can be tested. Bearing in mind that the hollow spaces might have had multiple origins, the following refutations may be only applied to the titanosaurs of Lo Hueco.

Given the extensive pneumatic features of the axial and appendicular skeleton of titanosaur dinosaurs^28^,^29^, it could be argued that some osteoderms might have been invaded by pneumatic tissue on occasion, hence the hollow spaces and the large trabeculae in HUE-08738 (Fig. 1). However, the camellae of titanosaur vertebrae have a smooth surface, which is absent in the canals and trabeculae of titanosaur osteoderms. This bony structure shows that when the pneumatic diverticula invaded the vertebrae, the destructive reabsorption was followed by the formation of a new bone surface in contact with the pneumatic epithelium. Also, the structure of the camellae has a honeycomb-like pattern, unlike the chaotic trabeculae from HUE-08738 (Fig. 1d). This suggests that the origin of the large trabeculae in the large hollow spaces of osteoderms is different from the pneumatic camellae, hence it is most likely that their formation is not related to pneumatic diverticula.

Also, most osteoderms with very dense spongy bone show evidence of a network of internal neurovascular canals (Fig. 2). The ramifications of these canals under the bulb and the pattern of development of the large hollow spaces, from the bulb toward the root end (Fig. 1c) show the formation of the large hollow spaces is directly linked to the main canals. This would be compatible with the hypothesis that mineral mobilization was the most likely cause of the hollow spaces of osteoderms.

Curry Rogers *et al*. interpreted the cavity in the large osteoderm FMNH PR 2342 from *Rapetosaurus* as osteoclastic remodeling in relation to blood calcium homeostasis^7^. They proposed several causes, among them adaptation to extreme seasonal changes, consequence of aging and eggshell formation during oogenesis. It was also suggested that recurrent remodeling under prolonged stress may have resulted in drastic changes in osteoderm morphology, perhaps becoming unrecognizable under prolonged stress and the consequent multiple reabsorptions (suggesting this might account for a preservational and/or collecting bias against titanosaur osteoderms in the Maevarano Formation)^7^. Considering that adaptation implies a selection pressure for a biological trait, if osteoderms were an adaptation to cope with extreme environments and/or seasonal changes, most individuals would mobilize minerals to cope with said changes. Therefore, the seasonal changes hypothesis predicts either a larger number of osteoderms with hollow spaces than those with dense bone or signs of mineral mobilization and later deposition, such as previous cavities filled with newly deposited bone (at least in the unfavorable season in an extreme environmental situation) or no hollow spaces at all (in a steady environment). If the main cause of large hollow spaces were processes related to aging, it would be commonplace in the older individuals among a population. Therefore, the aging hypothesis predicts that most of the osteoderms with internal hollow spaces would appear associated with senescent individuals.

Finally, the oogenesis hypothesis would predict both hollow and compact osteoderms of comparable larger sizes (attributable to adult reproductive female and male titanosaurs), and only compact “juvenile” osteoderms (such as UA 9331, Fig. 1c,d in Curry-Rogers *et al*.^7^). The proportion of hollow osteoderms would vary depending on multiple factors, such as preservation bias or adult sex ratios in the assemblage. Given that most reported adult sex ratios in modern birds and crocodile populations indicate parity or male biased ratios^30^,^31^,^32^, the expected number of hollow osteoderms should not be larger than half the sample.

Dermal bones of extant vertebrates are known to be a calcium source during oogenesis: the scales of elasmobranch and teleost fishes^33^ as well as living crocodiles. The osteoderms of the american *Alligator* have been found to mobilize at least 10% of their calcium in such periods^34^. On a study on 426 individuals of *Crocodylus johnstoni*, with 184 female crocodiles sampled, Tucker found that the oldest and largest females had extensive remodeling in their osteoderms^35^. Breeding females of *Crocodylus niloticus* also have been reported to extensively remodel and expand the vascular canals of their osteoderms^36^. Given that females of Alligatoridae and Crocodylidae living in different environments and continents subtract calcium from their osteoderms when gravid, the most parsimonious condition would be the same for their last common ancestor. Also, mineral reabsorption has always been reported as related to breeding extant crocodile females, whereas bone deposition (not decalcification) is always related to seasonal changes^35^,^36^.

Dacke *et al*. note that given their pneumaticity, the postcranial skeleton in titanosaurs may not have been enough to supply all the calcium needed during oogenesis, osteoderms might have supplied the calcium needed due their large, compact volumes^34^, as long bones might have been the original mineral source for osteoderm formation^37^.

The evidence found at Lo Hueco, which comes from bed G1 (Table 2), is a sample of most likely coexisting titanosaurs: G1 was the proximal part of a coastal flooded muddy plain with signs of fast accumulation of carcasses, such as over-representation of titanosaurs (96% of all dinosaur remains), absence of scavenging evidence or carcasses preferentially oriented^38^. Given that monodominant bonebeds are the product of catastrophic events in a short sedimentation timespan^39^ and the fact that all the titanosaur partial individuals are similarly scattered and articulated to similar degrees (Supplementary Fig. 1), the sample from G1 most probably records a single catastrophic event, in which the articulated remains accumulated once. The absence of scavenging marks on the bones, despite the presence of meat eaters in the site (theropods and crocodiles)^8^ and the absence of weathering in all the bones are also evidence of fast burial. Thus, the titanosaurs from G1 most likely lived in the same highly seasonal environmental conditions, with a thermal variability similar to that found today at the same subtropical latitudes (31°N) in similar environments, as determined by isotopic studies^40^. Preliminar analyses on histological samples of proximal shaft of dorsal ribs of HUE-EC-11, which was associated with the osteoderm HUE-00950 (Fig. 3, Fig. 10 of Vidal *et al*.^4^) show no evidence of the vast remodeling of overlapping osteons typical of senescent sauropods^41^,^42^.

The retrieved sample of three out of ten osteoderms with low-density bone in such conditions does not support the seasonal changes hypothesis (see Table 2) but would not refute the oogenesis hypothesis, as only a small fraction of the sampled osteoderms have hollow spaces or signs of past hollow spaces (not expected in the seasonal changes hypothesis, that is, as an adaptation to cope with extreme environments). Although the association of an osteoderm with a large internal hollow space with the non-senescent adult titanosaur HUE-EC-11 does not support mineral mobilization as a consequence of aging, the current evidence is probably not robust enough to refute the hypothesis. However, such an association implies that even if mineral mobilization occurred when aging, large hollow spaces originated from additional causes as well, as HUE-EC-11 is not histologically senescent (*sensu* Klein & Sander^41^).

The evidence from Lo Hueco is compatible with predictions from the oogenesis hypothesis: osteoderms of comparable sizes and morphology are both compact (HUE-01330) and hollow (HUE-00950) and, before mineral mobilization, they are the most readily available mineral reservoir in the titanosaur body (very dense spongy bone, Fig. 5). If supported by further evidence, the oogenesis hypothesis implies that the presence of hollow osteoderms might indicate its bearer was a gravid or nested female. Although circumstantial evidence, the fossil titanosaur eggshell bearing site of Portilla (Cuenca, Spain), also from the Villalba de la Sierra Formation^43^, is synchronous to Lo Hueco and is located about 30 km away. The fact that distantly related extant crocodile species mobilize calcium from their osteoderms when gravid implies that dermal armor is a readily source for labile calcium during oogenesis.

## Conclusions

The Upper Cretaceous site of Lo Hueco has one of the largest and better-preserved samples of titanosaur bulb and root osteoderms available from a single quarry and bed. This preservation has shown a consistent internal network of neurovascular canals, which enter the osteoderm from the basal foramen (under the bulb) and then branches laterally and in the antero-posterior axis. A small fraction of osteoderms from the G1 bed from Lo Hueco (three out of ten) have large internal hollow spaces, associated with this network of canals, implying mineral mobilization might be the cause of the hollows. The sample from Lo Hueco does not support the following hypotheses: (i) pneumatic origin of the large hollow spaces in osteoderms, and (ii) recurrent remodeling due mineral mobilization under prolonged stress as adaption to an extreme environment. However, the evidence from Lo Hueco cannot refute the hypotheses of mineral mobilization as a consequence of aging, occasional consequence of extreme environment/seasonal changes or as a part of the eggshell formation process. Finally, we conclude titanosaur osteoderms had an important physiological role, although the precise role is difficult to assess. The combination of several indirect lines of evidence, including population studies on extant armored archosaurs, support oogenesis as the most probable cause of mineral reabsorption in titanosaur osteoderms.

## Material

All the osteoderms studied in this paper belong to the bulb and root morphotype. Some of the authors described most of the sample in recent publications^4^,^16^. Ever since, new osteoderms have been found at the Lo Hueco site, in two beds (G1 and G2) out of the four sampled (see Barroso-Barcenilla *et al*. for geological context^19^). The new specimens show the same morphology and, when their silhouettes analyzed, they were retrieved within the morphospace delineated in previous publications. Thus, here we focus in describing the internal anatomy of some of the new finds, as the detailed anatomical description is beyond the scope of this work. The osteoderms studied are referenced in Table 1.

As for the anatomical terminology employed, and in order to keep it unambiguous and consistent with previous publications, we follow the terminology used by Cerda *et al*.^27^ instead of the one used by D’Emic *et al*.^3^ and Vidal *et al*.^4^, as the use of the terms “internal” and “external” could be misleading. The terminology is summarized in Fig. 1a. For a more in depth discussion on titanosaur osteoderm morphotypes see refs 3,4. The density of spongy bone described in the cores of the osteoderms through natural breakages is bimodal in osteoderms from Lo Hueco. That is, some osteoderms have very homogeneous and compact spongy bone (dense spongy bone, Fig. 5g; compact for short) and some osteoderms have a central cavity or void with sparse and with a chaotic architecture pattern trabeculae (large hollow spaces, Fig. 1b; hollow for short). The lower density areas seen in CT-scans are interpreted as large hollow spaces. DICOM files of the CT-scans of HUE-00950 (Fig. 3; Supplementary Data 1) and HUE-02000 (Fig. 4; Supplementary Data 2) are available as supplementary files.

## Methods

As some osteoderms were broken before or during excavation and preparation, the internal anatomy could be observed from the breakages. Also, a selection of titanosaur osteoderms from the sites of Lo Hueco and Armuña, with and without breakages, was CT-scanned at the Institut Català de Paleontologia Miquel Crusafont (Sabadell, Barcelona, Spain). After trying the most used voltage values for studying vertebrate fossils (120–180 kV)^44^, the inner structure of the osteoderms from Lo Hueco was indistinguishable, as they are filled with fine grain clays, with a density very similar to fossilized bone. Increasing the radiation voltage, the penetration power increased as well (in detriment of definition, with some ring artifacts appearing). The samples were irradiated with 450 kV voltage and 3.3 mA amperage. The inter-slice spacing was 0.33 cm. The scan data were imported into ImageJ 1.49b (National Institutes of Health, USA) for artefact removal and enhancing contrast. The enhanced data was imported to Avizo 7.1.0 (VSG, Burlington, MA, USA) and scrutinized, slice per slice, to thoroughly explore the internal microanatomy. From these slides, 3D models of the internal cavities of the osteoderms were created using the same software.

## Additional Information

**How to cite this article**: Vidal, D. *et al*. The internal anatomy of titanosaur osteoderms from the Upper Cretaceous of Spain is compatible with a role in oogenesis. *Sci. Rep.* **7**, 42035; doi: 10.1038/srep42035 (2017).

**Publisher's note:** Springer Nature remains neutral with regard to jurisdictional claims in published maps and institutional affiliations.

## Supplementary Material

Supplementary Figure 1

## Figures and Tables

**Table 1:** Titanosaur bulb and root osteoderms from Lo Hueco.

Collection number	Quarry bed	Large hollow spaces	Analysis
HUE 00561	G1	Yes	CT-scan
HUE 00590	G1	No	Natural breakages
HUE 00913*	G1	No	Natural breakages
HUE 00950	G1	Yes	CT-scan
HUE 01330	G1	No	Natural breakages
HUE 02000	G1	Yes	CT-scan
HUE 02029*	G1	No	CT-scan/Natural breakage
HUE 02326	G1	No	CT-scan
HUE 02900*	G1	No	Natural breakages
HUE 04505	G2	No	Natural breakages
HUE 08738*	out of context	Yes	CT-scan/Natural breakage
HUE 09393*	?	?	
HUE 02452	G1	No	Natural breakages
HUE 03767*	G2	?	
HUE 08665**	out of context	?	
HUE 01738**	G1	?	
HUE 07231	G2	No	Natural breakages

Broken specimens are marked with an asterisk (^*^). Too fragmentary specimens are marked with two asterisks (^**^).

**Table 2:** Hypotheses to explain the need for mineral mobilization in titanosaurs and how they cope with the evidence from Lo Hueco.

Hypothesis short-name	Low-density bone origin hypothesis	Predictions	Evidence-support
Pneumatic hypothesis	The low density bone of the osteoderms would be a by product of a pneumatization of the bone.	Bone similar to camellate bone.	N - the anatomical architecture of both types of bone is different.
Seasonal-changes hypothesis	The large hollow spaces of the osteoderms would result from mineral mobilization as an adaptation to extreme seasonal changes.	If the assemblage occured in an extremely seasonal environment, a majority of osteoderms should present large hollow spaces or signs of mineral mobilization (heavy remodeling and evidence of past hollow spaces)	N - osteoderms with low density bone are <50% of the sample which comes from an extremely seasonal environment. Past hollow spaces have not been identified either.
Aging hypothesis	The low density bone of the osteoderms would appear as a result of decalcification in older individuals.	Osteoderms with large internal hollow spaces associated with senescent individuals.	N - An osteoderm associated with large internal hollow spaces associated with a non senescent adult individual.
Oogenesis hypothesis	The low-density bone of the osteoderms is the result of the need of calcium for the formation of eggshells during oogenesis.	i) Osteoderms would be the only readily source for minerals in a titanosaur. ii) Osteoderms of comparable sizes (attributable to adult reproductive female and male titanosaurs) both compact and hollow. iii) Not a larger number of osteoderms with large hollow spaces (<50%), that is, those belonging to females in reproductive age.	Y - i) Osteoderms of comparable size classes from the same stratigraphic horizon are both hollow and compact. ii) A minority of osteoderms (3/10) have low density bone.

See discussion for a more in depth description of the hypotheses. Y = yes. N = no.
