# Supplementary material for: The internal anatomy of titanosaur osteoderms from the Upper Cretaceous of Spain is compatible with a role in oogenesis
Source: Sci Rep. 2017 Feb 7;7:42035. doi: 10.1038/srep42035 (PMC5294579; doi:10.1038/srep42035)

The internal anatomy of titanosaur osteoderms from the Upper Cretaceous of Spain is compatible with a role in oogenesis.

Daniel Vidal*[1,2], Francisco Ortega[1], Francisco Gascó[1], Alejandro Serrano Martínez[1] and José Luis Sanz[2]

1- Grupo de Biología Evolutiva, UNED, Paseo Senda del Rey 9, 28040, Madrid (Spain)

2 - Unidad de Paleontología, Universidad Autónoma de Madrid, C/ Darwin 2 28049, Madrid (Spain)

Supplementary Figure 1

Supplementary Figure 1.- General cartography of the G1 excavation area at Lo Hueco. Lines mark recognized partial titanosaurian skeletons. Yellow spots mark eight osteoderms. Two missing osteoderms from the map were collected ex-situ in G1, and therefore their precise location could not be assessed. Black spots mark isolated skeletal elements. This cartography was generated using Autodesk AutoCAD 2014 (http://www.autodesk.com/products/autocad/overview), from geo-referenced points measured in the field and the original quarry cartography drawn during the excavation.

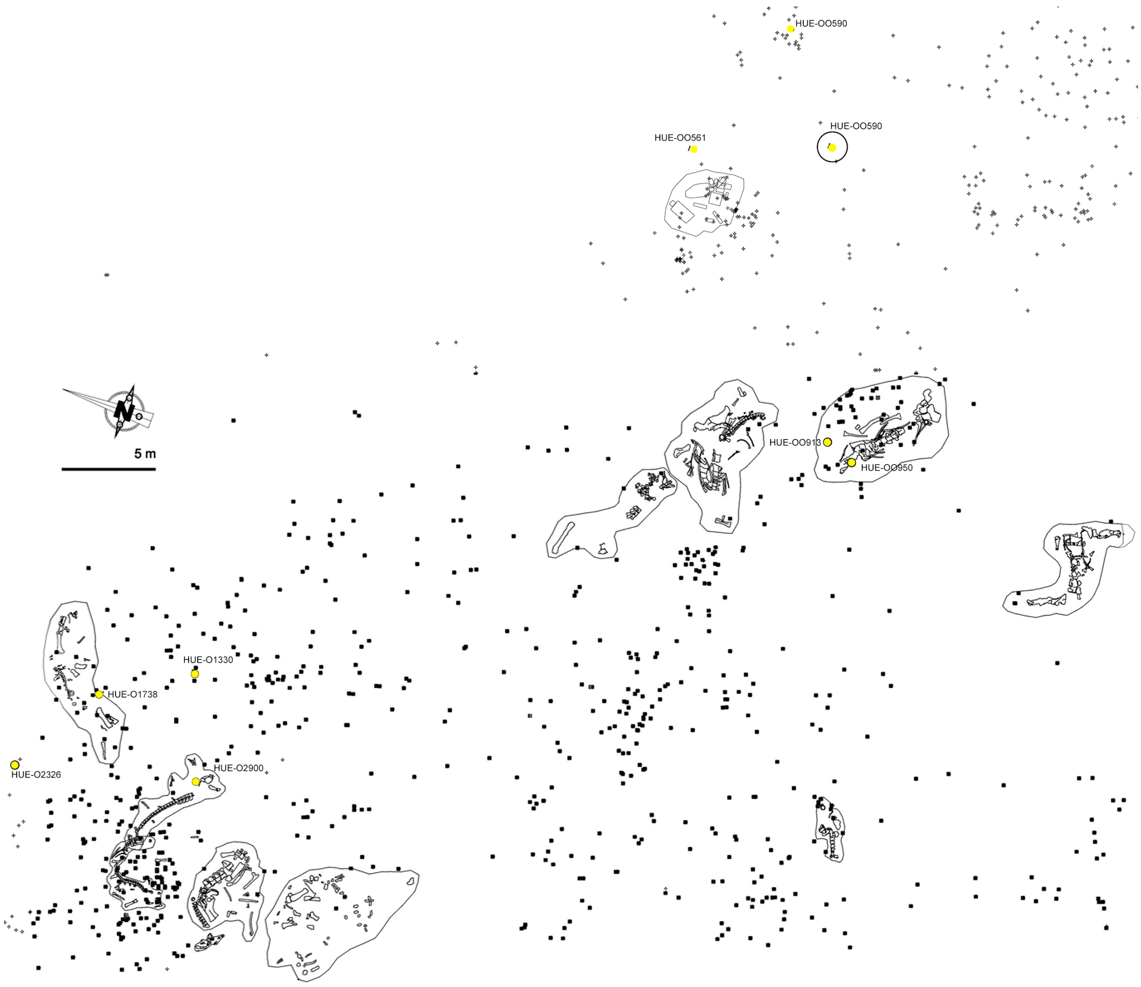

Supplement: Supplementary Figure 1 [file srep42035-s1.pdf]
